# Editorial: Bio-nanomaterials and systems for enhanced bioimaging in biomedical applications

**DOI:** 10.3389/fmolb.2024.1491376

**Published:** 2024-10-15

**Authors:** Sumin Park, Sudip Mondal

**Affiliations:** ^1^ Department of Biomedical Engineering, University of Michigan, Ann Arbor, MI, United States; ^2^ Digital Healthcare Research Center, Pukyong National University, Busan, Republic of Korea

**Keywords:** nanotechnology, bioimaging, molecular imaging, Raman spectroscopy, magnetic resonance imaging

## Introduction

This editorial provides an overview of the contributions to the Frontiers Research Topic “Bio-Nanomaterials and Systems for Enhanced Bioimaging in Biomedical Applications”.

Bioimaging includes a range of noninvasive techniques, such as X-ray, computed tomography (CT), magnetic resonance imaging (MRI), single-photon emission computed tomography (SPECT), surface-enhanced Raman spectroscopy (SERS), fluorescence microscopy, photoacoustic microscopy, scanning acoustic microscopy (Lahoti et al.). These techniques allow for the visualization of an organism’s structure and the clarification of various biological functions in real-time, using two and three-dimensional images. Significantly, bioimaging achieves this without causing any physical disruption, such as movement or respiration. It facilitates observation across several levels of cellular organization, including subcellular structures, tissues, and organisms, providing valuable insights into physiological and pathological processes (Wallyn et al., Raghavendra et al.). Bioimaging enhances our understanding of complex biological systems and contributes to human welfare by improving the accuracy of diagnosis, the effectiveness of treatment, and the prevention of disease (Hussain et al.). Furthermore, it improves clinical research by developing and improving more sophisticated imaging techniques, leading to significant advances in safety and practical applications.

Moreover, bio-nanomaterials play a crucial role in enhancing bioimaging by improving the specificity, sensitivity, and multifunctionality of imaging contrast agents. These materials, including nanoparticles, quantum dots, and nanostructured materials, are engineered to interact with biological systems at the molecular level (Hsu et al., Liu et al.). This allows for more accurate targeting and visualization of biological processes. By integrating bio-nanomaterials with existing imaging techniques, researchers can achieve higher-resolution images, deeper tissue penetration, and more accurate diagnoses, ultimately advancing the field of biomedical imaging.

Therefore, this Research Topic brings together cutting-edge research on the integration of bio-nanomaterials with bioimaging systems. This presents an overview of bio-nanomaterials and systems for improved bioimaging and offers prospective future avenues for bioimaging.

## Summaries of contributed articles

Li et al. utilized *clMagR/clCry4* gene as an MRI T2-contrast agent, demonstrating its ability to confer MRI properties to living *Escherichia coli* (*E. coli*) in the presence of Fe^3+^. This gene transfection significantly enhanced the uptake of exogenous iron by *E. coli*, leading to the formation of iron oxide nanoparticles through intracellular co-precipitation, which could mediate the alteration of the MRI transverse relaxation rate. Experimental results showed that the pure *clMagR/clCry4* protein exhibited little MRI contrast effect, thus its transfection was unable to influence MRI spin relaxation signals. Therefore, they hypothesized that the magnetism of the *clMagR/clCry4* protein would be associated with the electron transfer. The experiments to verify this proved that *clMagR/clCry4* transfected *E. coli* exhibited significant MRI-T2 contrast after culturing with ammonium ferric citrate (FAC) as an exogenous iron donor, while having minimal impact on MRI-T1 mode. The study also revealed that increasing iron levels or bacterial density resulted in a more pronounced T2-contrast effect in the transfected *E. coli*. These findings not only present a novel approach for using genetic modification and developing live contrast agents but also open up new avenues for exploring *clMagR/clCry4* gene in biological imaging applications. Furthermore, due to the role of *clMagR/clCry4* protein in magnetoreception, this study is useful for harmonizing the long-term controversy over the existence of magnetoreceptors in organisms, ranging from prokaryotes to animals.

The article by Wang et al. presents a promising strategy for improving the pharmacokinetic profile of artesunate (ATS) through exosome-drug conjugates (EACs). The research is well-detailed, showcasing significant enhancements in ATS solubility, bioavailability, and sustained release when delivered via milk-derived exosomes. The methodology is robust, with thorough characterization of EACs and comparative analyses against traditional drug delivery methods. However, the article could benefit from a clearer discussion of the potential challenges in scaling up this technology for clinical use, particularly regarding the production and standardization of exosomes. Additionally, while the study is comprehensive, it might overwhelm readers with technical details; a more concise summary of key findings would improve its accessibility. The implications of this research are significant, but the article should also address potential regulatory hurdles and long-term safety concerns of using exosomes in drug delivery. Overall, the study offers valuable insights into exosome-based drug delivery, but further exploration of its practical application and broader impact is needed.

Xie et al. provided a comprehensive overview of the latest developments, challenges, and potential prospects in GLP-1R molecular imaging using radiology theranostics techniques such as PET, CT, and SPECT. GLP-1R, also known as glucagon-like peptide-1 receptor, is an important target in various medical applications because of its significant role in glucose metabolism and insulin secretion. Radiology theranostics is a cutting-edge approach to tailored medicine that combines diagnostic imaging and therapeutic interventions. It allows for accurate illness characterization and targeted treatment. The integration of GLP-1R molecular imaging and radiology theranostics shows potential for improving the accuracy of diagnosis and effectiveness of treatment in various conditions, including insulinomas, neoplasms, myocardial ischemia, endocrine-related fields including beta-cell mass monitoring, islet transplantation, congenital hyperinsulinism, multiple endocrine neoplasia type 1, and neurodegenerative diseases. Utilizing molecular probes such as exendin-4 has demonstrated the ability to enhance imaging results by offering a high level of accuracy and sensitivity in detecting GLP-1R expression. However, the field encounters challenges such as the limited diversity of accessible imaging agents and the requirement for additional investigation to optimize probe design and clinical applications. Notwithstanding there are limitations, the development of more all-encompassing GLP-1R molecular imaging agents may significantly improve theranostic capabilities, offering new opportunities for disease monitoring and management. These researches emphasize the significance of ongoing exploration and innovation in this field to overcome current hurdles and fully achieve the potential of GLP-1R-based theranostics through the use of radiological imaging techniques.

Another study Chen et al. provides a comprehensive review of the applications of Raman spectroscopy in diagnosing and monitoring neurodegenerative diseases, emphasizing its potential as a non-invasive and highly sensitive diagnostic tool. The discussion is thorough, exploring various aspects of Raman spectroscopy, from its principles to its application in specific diseases like Alzheimer’s and Parkinson’s. However, the article could benefit from a more focused narrative, perhaps by narrowing the scope to fewer neurodegenerative diseases for deeper analysis. The inclusion of more recent case studies or clinical trial data could enhance its practical relevance. Additionally, the article’s length and density may overwhelm readers who are not specialists in the field; a more concise summary of key findings and future prospects could improve accessibility. Finally, while the article is scientifically rigorous, it might benefit from more critical analysis of the limitations and challenges in translating Raman spectroscopy from research to clinical practice.

## Conclusion

The contributions to this Research Topic underscore the critical significance of bio-nanomaterials in enhancing biomedical imaging. The works in this Research Topic showcase notable progress in biomedical imaging achieved by employing bio-nanomaterials. However, they also emphasize the crucial challenges that need to be addressed, such as biocompatibility, targeted delivery, and reproducibility across biological systems. These challenges are pivotal for translating innovations into clinical practice. However, the contributions highlight the significant impact that bio-nanomaterials can have on improving imaging applications. This opens possibilities for non-invasive, theranostics, and personalized medicine advancements as the integration with advanced imaging systems progresses.

